# The impacts of and outcomes from telehealth delivered in prisons: A systematic review

**DOI:** 10.1371/journal.pone.0251840

**Published:** 2021-05-17

**Authors:** Esther Jie Tian, Sooraj Venugopalan, Saravana Kumar, Matthew Beard

**Affiliations:** 1 Allied Health and Human Performance Unit, University of South Australia, Adelaide, South Australia, Australia; 2 Spinal Assessment Clinic, Royal Adelaide Hospital, Adelaide, South Australia, Australia; 3 Spinal Assessment Clinics, Spinal Unit Office, Royal Adelaide Hospital, Central Adelaide Local Health Network, Adelaide, South Australia, Australia; University of North Carolina at Chapel Hill, UNITED STATES

## Abstract

**Background:**

While the delivery of healthcare services within prison systems is underpinned by different models, access to timely and optimal healthcare is often constrained by multifaceted factors. Telehealth has been used as an alternative approach to conventional care. To date, much of the focus has been on evaluation of telehealth interventions within certain geographical contexts such as rural and remote communities. Therefore, the aim of this systematic review was to synthesise the evidence base to date for the impacts of, and outcomes from, telehealth delivered in prisons.

**Methods:**

This systematic review was underpinned by best practice in the conduct and reporting of systematic reviews. A systematic search was conducted to reinforce the literature selection process. The modified McMaster Critical Appraisal Tool was used to assess the methodological quality of the included studies. A narrative synthesis of the study outcomes was undertaken.

**Results:**

Twenty-nine quantitative studies were included. Telehealth interventions were greatly varied in terms of types of healthcare services, implementation process and intervention parameters. Methodological concerns such as rigour in data collection and analysis, and psychometric properties of outcome measures were commonly identified. *Process-related outcomes* and *telehealth outcomes* were the two overarching categories identified.

**Conclusion:**

This systematic review provides mixed evidence on the impact of, and outcomes from, telehealth in prisons. While the evidence base does highlight some positive impacts of telehealth, which at the least, is as effective as conventional care while achieving patient satisfaction, it is also important to consider the local context and drivers that may influence what, when and how telehealth services are provided. Addressing critical factors throughout the lifecycle of telehealth is equally important for successful implementation and sustainability.

## Introduction

As the prison population continues to grow worldwide [1], so does the spectrum and severity of health issues experienced by prisoners when compared to the general community [2]. For example, in Australia, about half of prison entrants (49%) report a mental health disorder history diagnosed by a doctor, psychiatrist, psychologist or nurse, with more than one in four (27%) receive medications for mental health disorder management [3]. The high prevalence of mental health issues amongst the incarcerated population is not limited to Australia, in fact, it is a widely recognised problem across multiple countries such as New Zealand, North America and within Europe [2, 4]. A previously published review suggests that approximately one in seven prisoners in Western countries experience psychotic illnesses or major depression, which are possible risk factors for suicide. This review further indicates that prisoners are about ten times more likely to be diagnosed with antisocial personality disorder, compared to the wider population [4].

In addition to mental health disorders, the prevalence of communicable diseases, particularly blood borne viruses and sexually transmissible infections (STIs), is substantial in prisons. International evidence has demonstrated remarkably higher rates of the Human Immunodeficiency Virus (HIV) infection amongst prisoners than the general population [2, 3] with some research estimating it to be as high as 10% [3, 5]. Similar higher rates for chlamydia, gonorrhoea, syphilis, Hepatitis B and Hepatitis C have also been reported [6, 7]. Non-communicable diseases (NCDs) are also over-represented in the prison system [8]. In Australia, almost a third of prisoners report history of one or more chronic conditions including asthma, arthritis, cardiovascular disease (CVD), diabetes, or cancer [9]. Prisoners in the United States (U.S.) also experience a higher prevalence of NCDs such as hypertension, asthma, arthritis and cervical cancer, with odds ratios ranging between 1.2 and 1.8 compared to their community counterparts [10]. Similar findings have been reported in Spanish prison population where one in every two prisoners has some type of chronic disease [11], while a recent systematic review highlights that prisoners aged 50 years and over often face a higher burden of NCDs, as opposed to younger prisoners or their aged-matched community peers [12].

The delivery of healthcare services in prison systems is underpinned by various models, ranging from healthcare provided by employees within the prison system to services delivered through community-based systems or by local contracted health professionals [2, 13]. In Australia, the delivery of prison healthcare services is different in many aspects compared to the wider community. In particular, the primary healthcare is primarily delivered by nurses in prisons; whereas in the general community, general practitioners (GPs) are usually the first level of contact [3, 14]. Depending on the custodial setting, jurisdiction and healthcare required, specialist care including secondary, tertiary and allied healthcare services are either provided within the prison system or off-site through community-based services, such as emergency care and general hospital inpatient [3, 14].

Despite the diverse service delivery models in prisons, access to timely and needed healthcare is often challenging. A recent report from the Australian Institute of Health and Welfare highlights that only 50 per 1,000 inmates received treatment for Hepatitis C [9], in spite of a noticeable increase from eight per 1,000 in 2015 [3]. Factors contributing to limited access to healthcare services during incarceration are complex and multifaceted. These include individual-related barriers such as lack of understanding about the disease and the effects associated with treatment [15] and lack of knowledge regarding existing health services in prison [15, 16]; organisation-related issues such as delays in approval of healthcare [15, 16] and limited patient-centred approach due to security restrictions [17]; and social/cultural factors such as stigma and concerns about confidentiality [15]. Costs associated with transportation as well as potential risk of prison escapes further hinder the provision of timely and optimal healthcare services [2, 18].

Telehealth has been used as an alternative approach to conventional care in a range of healthcare disciplines as means of reaching populations with poor access to healthcare services, such as rural and remote communities [19]. The term *telehealth* is defined as the delivery of health services at a distance with the use of information and communication technologies [20]. It is a broader term which encompasses patient/ professional education and administration, in addition to telemedicine (which is more specific to provision of clinical services), although it is often used interchangeably with the term *telemedicine* in the literature [20, 21]. Such approach may use videoconferencing for real-time consultations (synchronous modality) or ‘store-and-forward’ technologies for transference of medical data such as images, notes and diagnostic test results, which are later reviewed by health providers for diagnosis and management (asynchronous modality) [21].

The historical utilisation of telehealth within correctional institutions is well-documented, particularly in the U.S. [22, 23]. However, the implementation of telehealth across prison services has been less extensive elsewhere, such as in Australia. While there is growing evidence to support telehealth as a model of care for those who are disadvantaged by location or circumstances from receiving healthcare services, the literature has largely focussed on rural and remote communities [24–26]. Therefore, the aim of this systematic review was to synthesise the evidence base to date for the impacts of, and outcomes from, telehealth delivered in prisons.

## Methods

### Search protocol and registration

A protocol for this systematic review was registered with PROSPERO (CRD42018093766) and has since been published online.

### Literature search

Electronic database searches of Ovid (MEDLINE; Embase; Emcare), Educational Resources Information Centre (ERIC), the Cochrane Library, PsycINFO, Joanna Briggs Institute, ProQuest, Social Care Online, National Institutes of Health, EBSCOhost (Academic Search Premier; Australia and New Zealand Reference Centre), Informit (health; Indigenous; social sciences), Scopus and Web of Science were initially performed between 9–11 May 2018 to capture relevant articles. The development of search strategy was underpinned by three concepts–Telehealth; Prison; Impact and Outcomes. The key search terms and Medical Subject Headings (MeSH) relevant to each concept were used during the literature searching process, with application of relevant truncations and Boolean operators (Table 1). For the purpose of this review, the terms telehealth and telemedicine were used synonymously to (i). ensure a comprehensive coverage of all relevant literature as these terms were used interchangeably and (ii). reach a broad audience who may use either term. The three concepts were later combined (using AND) to allow for the most number of relevant articles to be captured. A full search strategy in Ovid Medline is presented in S1 Table.

**Table 1:** Search strategy.

Concepts	Key search terms	Medical Subject Headings (MeSH)
*Concept One: Telehealth*	telehealth or telemedicine or ehealth or mhealth or telepatholog* or teleradiolog* or telerehabilitat* or teleconsult* or telecare or telemonitor* or telepsychiatr* or teleaudiolog* or telespeech* or teleaudiometr* or e-health or m-health or videoconferenc* or video-conferenc* or video-monitor* or videomonitor* or videoconference* or “(rehabilitat* or consult* or health or pathology* or radiolog* or medicine or care or monitor* or psychiatr* or audiolog* or speech or audiometr*) adj5 (virtual or remote or tele or mobile)”	“telemedicine/” or “telerehabilitation/” or “teleradiology/” or “telepathology/” or “remote consultation/” or “videoconferencing/”
*Concept Two: Prison*	confined or confinement or imprison* or inmate* or incarcerat* or jail* or gaol* or “(penal or custodial or correction or detention) adj5 (institution* or facility* or center$1 or centre$1)”	“prisons/” or “prisoners/”
*Concept Three: Impact and Outcomes*	assess* or outcome* or measure* or eval*	“outcome and process assessment (health care)/” or “outcome assessment (health care)/”

As means of avoiding publication bias, and to improve the overall reach and spread of this systematic review, a grey literature search was also undertaken on 11 May 2018 using an internet web engine (Google and Google Scholar) to capture any additional publications such as governmental and technical reports. Furthermore, reference lists of the included articles were searched to maximise the retrieval of relevant publications (pearling). An updated search of all but two of the aforementioned electronic databases, Google and Google Scholar was performed between 4–6 February 2020 to capture any newly published and eligible articles. Updating the searches in Academic Search Premier and Australia and New Zealand Reference Centre was restricted as access to these two databases was no longer available through the institution. All searches were limited to English language but no restrictions on publication dates.

### Study selection

Primary quantitative research studies (including both experimental and observational studies) were included if they had telehealth intervention delivered by any health professional and involved a consultation that aimed to provide healthcare assessment and management to people of all ages and genders in prison/correctional services, and studies discussed patient and clinical outcomes (including but not limited to patient satisfaction, function, quality of life, activity and participation levels, emotional well-being) or health service outcomes (including but not limited to cost savings, access to care, ease of delivery, continuity of care and time saving). Exclusion criteria were studies which (i). included telehealth interventions that used ‘store-and-forward’ technology for information (as the asynchronous approach may not involve consultations between a health professional and a patient) or remote in-home monitoring alone with no consultation or service provision, (ii). used real-time teleconsultation as part of an intervention but did not evaluate its effectiveness separately, (iii). included participants who were refugees, on parole or post-release from a correctional facility or people undergoing home detention, or (iv). non-English language literature, secondary research (such as structured literature or systematic reviews), qualitative research studies, descriptive cross-sectional studies (such as outcome measures solely focussed on the number of consultations or patients), protocols, editorials, conference proceedings, opinion pieces or commentaries.

The titles generated by the electronic databases were scanned to identify potentially relevant papers and where the titles would not allow for determination of relevance to the topic, abstracts were reviewed. If the titles and abstracts met the inclusion criteria for this review, they were initially selected to be part of the review. Full-text copies of eligible articles were later retrieved for full examination. During this process, the complete papers were examined to identify if they met the inclusion criteria for this review. Publications, which met all the inclusion parameters, were included in this review. Two reviewers (ET and SK) independently screened a proportion of identified citations (approximately 10%), due to resource constraints, and only after establishing consistency between the reviewers, the remaining citations were screened by one reviewer (ET). The literature selection process was completed in accordance with the Preferred Reporting Items for Systematic Reviews and Meta-Analyses (PRISMA) criteria [27].

### Assessment of study quality

The methodological quality of included studies was evaluated using a modified version of McMaster Critical Appraisal Tool (CAT) for quantitative studies [28]. The modified McMaster CAT was chosen for its generic nature by design (that is it is not specific to individual research designs) and as such can be used across multiple research designs. The research team has previously used this tool successfully across a number of systematic reviews [29–32].

The assessment criteria were grouped into eight categories: study purpose, background literature review, study design, sample, outcome measures, intervention, results, and conclusions and clinical implications. A total of 14 applicable criteria were available for assessment. Each criterion required a ‘yes’, ‘no’, ‘not addressed’, or ‘not applicable’ response. A scoring system was employed, where each ‘yes’ would receive one point, each ‘no’ or ‘not addressed’ would score as zero and ‘not applicable’ would reduce the total number of applicable criteria. Studies were not excluded based on their methodological quality. However, this information was used to report, analyse and discuss the overall review findings.

### Data extraction and synthesis

A customised data extraction form was developed specifically for this systematic review. The form contained key elements including author(s), year of publication, country of origin, study design, sample size, characteristics of study participants and/or settings, data collection methods, intervention description and key outcome domains as pertinent to address the objectives of this systematic review. Study findings relevant to impacts and outcomes regarding the delivery of telehealth in prisons were also documented.

A narrative synthesis of the study outcomes was performed in this systematic review. Meta-analysis of the included studies was not undertaken for a number of reasons. First, there was a great deal of heterogeneity given the variability of the evidence base (study designs, interventions administered and outcomes measures). Second, where there was consistency, such as in outcomes, it was limited to a handful of studies. Finally, when meta-analysis was attempted, heterogeneity values (such as I^2^) indicated substantial heterogeneity, which called into question the appropriateness of undertaking a meta-analysis. Instead, narrative synthesis is considered as one of the best approaches for synthesising findings from multiple studies when statistical meta-analysis or another specialist approach is deemed not suitable due to heterogeneity [33]. By using a textual approach to summarise and describe the findings, narrative synthesis ‘tells the story’ of concerned issues [33]. Given the aim of this systematic review was to investigate the impacts of, and outcomes from, telehealth delivered in prisons and the existence of heterogeneity across the included studies, narrative synthesis was chosen to underpin the understanding of the issue. Relevant data were extracted and synthesised by one reviewer (ET), with ongoing consultation regarding uncertainties with a second reviewer (SK).

## Results

**Fig 1:**
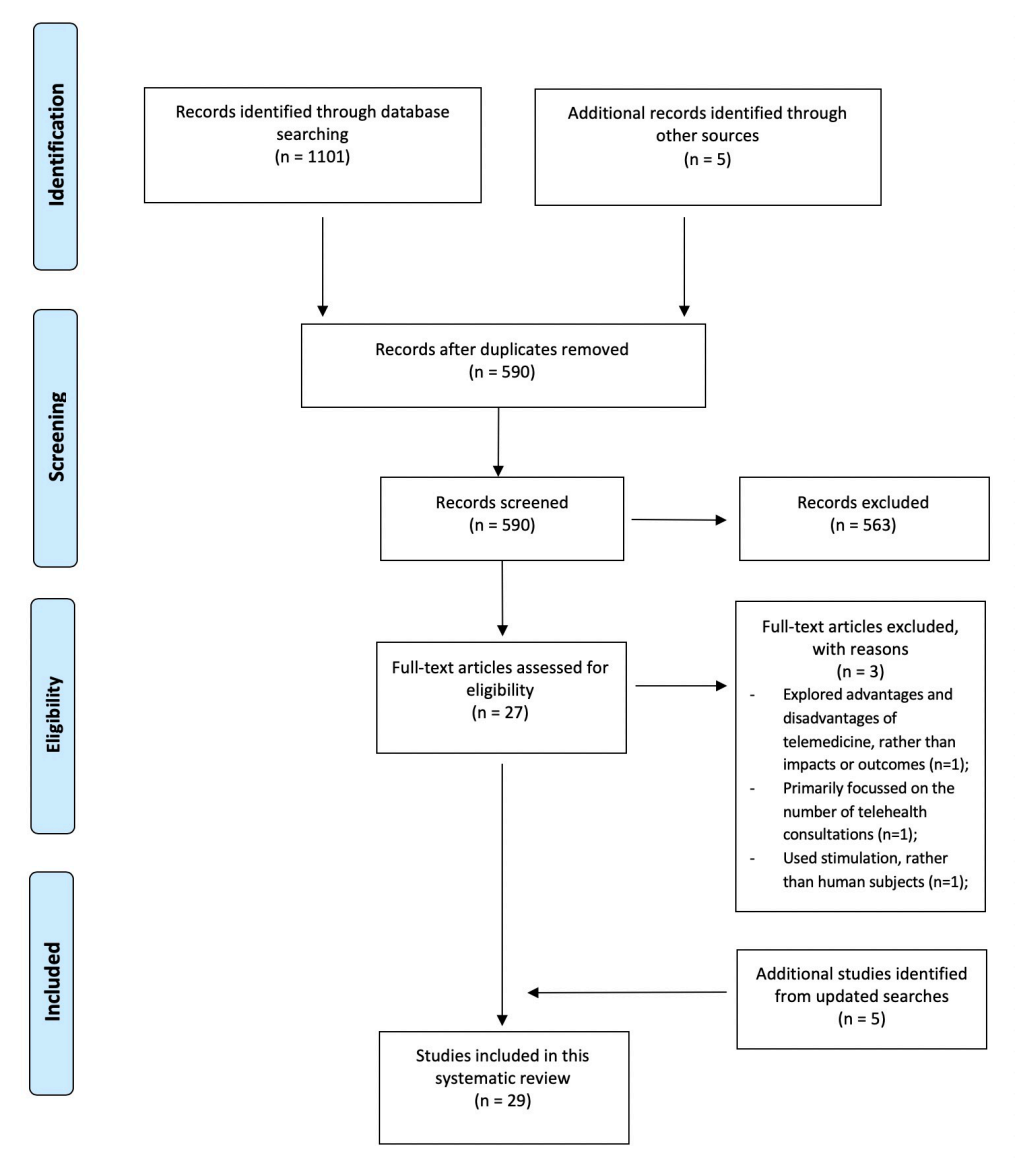
PRISMA flow chart.

The literature search yielded a total of 1106 records, including 1101 records from electronic databases and an additional five studies were identified from grey literature search and pearling. After removing 516 duplicates, 590 articles were screened for title and abstract relevance. Another 563 articles were further excluded after screening as they did not meet the inclusion criteria for this review. Of the remaining 27 studies obtained in full-text, three studies were excluded as they (i).explored advantages and disadvantages of telemedicine, rather than impacts or outcomes (n = 1); (ii).primarily focussed on the number of telehealth consultations (n = 1); (iii).used stimulation, rather than human subjects (n = 1). Additionally, another five studies identified from the updated searches were further included. Overall, a total of 29 studies met the inclusion criteria and therefore were included in this systematic review (Fig 1).

### Study characteristics

All studies included in this review were quantitative research studies, including both experimental and observational designs. The data collection methods ranged from retrospective collection of relevant data (such as review of historical records) to prospective data collection (such as completion of a questionnaire at post consultation). Majority of the included studies (n = 24) were undertaken in the U.S. [19, 34–56], with one each in Australia [57], China [58], South Korea [59], Spain [60] and the United Kingdom (U.K.) [61]. All studies were published between 1996 and 2019.

Adolescents and adults aged between 12 and 76 years were included, with males being the predominant focus across the included studies. The sample size varied from 43 to over 4000 subjects. The types of correctional facilities were different in aspects of security requirements (ranging from minimum to maximum security), location (ranging from rural to urban location) and housing capacity. There was also a great deal of diversity regarding telehealth interventions, especially in terms of healthcare services (such as psychiatric services versus urological care), implementation process (such as on-site teleconsultation versus involvement of some transportation, presence versus absence of prison staff during consultation) and intervention parameters (such as session duration, frequency of consultation). A summary of study characteristics is presented in Table 2.

**Table 2:** Study characteristics.

Study, year & Country	Study Design	Sample Size	Characteristics of participants and settings	Data Collection Methods	Intervention	Key outcome domains
Batastini & Morgan 2016 [34] U.S.	Quasi-experimental	n = 49 segregated inmates VC = 24; in-person = 12; no-treatment control = 13	***Age*:** mean = 29.8 years (SD = 8.2); ***Gender*:** all males; ***Ethnicity*:** 44.9% White; ***Medical conditions*:** primary substance-use (38.8%) or mood-related disorder (26.5%); ***Others*:** an average of 5.2 years (SD = 1.8) in prions on current conviction(s); duration in special management housing ranged from less than a year to 11.4 years (average = 1.3 years; SD = 1.8); 11.4 years (SD = 1.4) of formal education;	Electronic records database and participants’ self-report;	Psychotherapy intervention–Coping skills group: • Delivered by a Master’s -level mental health provider; • Weekly one-hour group sessions over 6 weeks; • Sessions delivered through: ◦ VC with 4 inmates or less per group (maximum of 6); ◦ In-person with 2 inmates per group;	Clinical-related outcomes: *Pre-post difference* • Mental health functioning (SCL90-R); • Criminal thinking (PICTS and MOCTS); • Frequency of inmate disciplinary infractions and mental health-related sick call requests; Process-related outcomes: *VC and in-person groups only; post treatment* • Level of satisfaction of patients (CSQ-8); • Work alliance (WAI); • Intervention effectiveness (I-GES); • Treatment completion status;
Brodey et al 2000 [35] U.S.	Non randomised control	n = 43 forensic psychiatric inmates In-person = 20; telepsychiatry = 23	**Total** ***Age*:** ranged 20 to 57 years; ***Medical conditions*:** 72% (n = 31) had medications prescribed, including antidepressants (48%; n = 15), mood stabilisers (39%; n = 12), antipsychotics (23%; n = 7) and anxiolytics (13%; n = 4); ***Setting*:** a large urban jail; **Telepsychiatry group** ***Age*:** mean = 36.3 years (SD = 9.3); ***Global Severity Index***: mean = 1 (SD = 0.3); **In-person group** ***Age*:** mean = 31.8 years (SD = 9.6); ***Global Severity Index***: mean = 0.9 (SD = 0.2);	Completion of a patient satisfaction survey (the Group Health Association of American Consumer Satisfaction Survey) by participants immediately after an evaluation;	Psychiatric evaluations (in-person or remotely through telemedicine) were conducted on alternating weeks over a 10-week period by the same psychiatrist;	Level of satisfaction of patients;
Brunicardi 1998 [36] U.S.	Case report	n = 469 telemedicine consults	NR	Relevant data were collected from the following sources: • The Bureau of Medical Service position control roster; • Interviews with personnel from the institutions; • Written logs from the Corrections Medical Centre and the institutions;	Implementation of telemedicine services;	Costs comparison between telemedicine and traditional modes of care delivery;
Cheng et al 2018 [58] Hong Kong, China	Case control	Intervention: n = 86 Control: n = 249	**Total** ***Age*:** ranged 21 to 64 years ***Gender*:** all males; **Intervention** ***Age*:** 40.4±11.0 years ***Principal diagnosis*:** 52.3% (n = 45) substance abuse, 27.9% (n = 24) schizophrenia, 5.8% (n = 5) affective disorder and 14% (n = 12) others; **Control** ***Age*:** 40.2±9.2 years ***Principal diagnosis*:** 51.8% (n = 129) substance abuse, 15.7% (n = 39) schizophrenia, 7.2% (n = 18) affective disorder and 25.3% (n = 63) others; *Statistically significant difference in the principal diagnosis between the two groups (p =* .*029)*	Completion of C-GHQ-12 by both groups within 7 days before and after the consultation; Completion of a satisfaction questionnaire (in Chinese) by participants from the intervention group;	Psychiatric teleconsultations: • Patients were transferred to a reception centre for teleconsultations; • A qualified nurse from the correctional site was present during the teleconsultation with the patient; • The mean duration of the teleconsultations was 6.33±3.58 minutes; • A maximum of 4 consecutive teleconsultations were allowed for the intervention participants;	The effect and sustainability of the effect of teleconsultations, in terms of: • Pre-post difference in C-GHQ-12 score of both groups; • Pre-post difference in C-GHQ-12 score of the first and second teleconsultations; Level of satisfaction of patients; Number of adverse events;
Fox, Somes & Waters 2006 [37] U.S.	Pre-post, quasi-experiment	n = 4 YDCs	***Setting*:** rural and underserved areas; housing youths aged between 12 to 19 years; predominantly males; 55% were Black;	Relevant data were collected from medical bill, facility travel logs and records;	Implementation of telemedicine services as part of the Kids in Custody Program;	Cost-effectiveness of telemedicine program: •Cost per person per month by site: ◦ ER visits; ◦ Specialty consults; ◦ Inpatient care; ◦ Transportation and security; • Impact of telemedicine utilisation on cost measures;
Fox, Somes & Waters 2007 [38] U.S.	Pre-post, quasi-experiment	n = 4 YDCs	***Setting*:** average number of housed adolescents ranged between 109 to 139; average age ranged between 16.24 to 17.5 years; 83% - 100% males; 55% African-Americans;	Retrospective data collection from medical records, assessment logs, claims data, and travel logs;	Telemedicine program: • Over half of the consultations involved behavioural healthcare services; • The on-site clinical nurse presented patients via telemedicine to the remote physician, using the local telemedicine equipment to transmit information and reporting any other physical findings; Consultations were conducted between the patient and the paediatric psychiatrist during behavioural healthcare visits;	Timeliness of care delivery; *Behavioural healthcare only* Access to healthcare services, including outpatient, ER and inpatient visits:
Fox et al 2008 [39] U.S.	Pre-post	n = 190 subjects from n = 3 YDCs	***Age*:** average = 16.7 years; ***Setting*:** housing youths aged between 12–19 years (average = 17 years); predominantly males; 55% African-Americans; 64% physically violent offences and 22% sexually violent crimes; average daily census between 350 and 369	Retrospective review of documents;	Telemedicine program: • A nurse from the detention facilities presented the patient during telemedicine encounters; • Including psychiatric counselling services and other services e.g. dermatology and ENT;	IPP goal establishment and attainment;
Jameson et al 2008 [40] U.S.	Observational study	n = 43 inmates	***Age*:** mean = 43.7 years (ranged between 22.3 and 68.9 years); ***Gender*:** all males; ***Medical conditions*:** type I (35%) or type II diabetes with a mean duration of 16.5 years (ranged 1–51 years); had no or various comorbidities; therapy consisted of insulin and/or oral agents; ***Setting*:** from n = 12 state penal institutions;	Retrospective chart reviews	Telemedicine for management of diabetes: • Lab results, physical exam and medical information were faxed to the endocrinologist before televisits; • Monthly telemedicine consultations by the endocrinologist; • Follow-up visits were suggested at 3- to 4-months intervals; • All initial and follow-up consultations were conducted through telemedicine;	Impact of telemedicine on glycaemic control;
Jimenez-Galan et al 2019 [60] Spain	Observational study	n = 163 inmates	***Age*:** 46.7±8.16; ***Gender*:** all males; ***Medical conditions*:** 57.7% (n = 94) HCV mono-infection and 42.3% (n = 69) HCV-HIV co-infected; varied hepatic function, stage of liver fibrosis and virological characteristics; ***Setting*:** a large penitentiary institution houses 1,200 inmates;	Blood samples, transient elastography and ultrasound scan; Completion of a satisfaction survey (on technical quality of image and sound, comfortability, perceived professionalism and overall assessment) by the inmates and participating doctors at one-year post implementation;	Telemedicine integrated into the entire HCV healthcare process: • Including screening, clinical evaluation, indication of treatment, on-treatment monitoring, follow-up and education to prevent reinfection; • Conducted with a doctor and a nurse at the correctional facility, in collaboration with the specialist at the hospital site; • The clinical inspection was performed with the camera and close-up camera and the physical exploration was conducted by doctors at the correctional facility; • The gastroenterology and infectious diseases consultation once a week; • An average of 4 patients were seen each week;	Effectiveness of integrating telemedicine into the HCV healthcare cascade, in terms of: • Overall SVR rate; • Rescue of patients with virological failures and number of reinfection cases; Level of satisfaction of participating inmates and doctors;
Kassar, Roe & Desimone 2017 [41] U.S.	Observational study	n = 106 inmates	***Age*:** mean = 43.7 years (SD = 12.75); ranged between 20 and 75 years; ***Gender*:** all males; ***Medical conditions*:** type I (44.3%) or type II (55.7%) diabetes with an average duration of 19.7 and 10.9 years, respectively; 96 were treated with insulin; ***Setting*:** from n = 15 correctional facilities;	Retrospective chart reviews	Implementation of telemedicine for management of diabetes: • Recent lab results (HbA1c and lipid panels), imaging studies, medication list, allergies, and problem lists were faxed to the endocrinologist prior to each televisit; • Presence of a prison nurse with the patient for each televisit; • Vital signs were collected by a prison nurse and verbally reported to the endocrinologist during the televisit; Follow-up visits were recommended within 2–4 months;	Impact of telemedicine on diabetes management in areas of: • Glycaemic control; • Blood pressure measures; • LDL levels;
Magaletta, Fagan & Peyrot 2000 [42] U.S.	Observational study	n = 75 inmates	***Gender*:** all males; ***Medical conditions*:** based on their diagnoses, they were grouped into "thought disorder" category (n = 17); "affect disorder" (n = 26); and "other (e.g. personality disorders, substance abuse disorders)" (n = 32);	Completion of a six-item questionnaire (developed by the psychologists) after each telehealth consultation;	Implementation of telehealth pilot program for mental healthcare: • Consultations were conducted by a psychiatrist remotely, with presence of the inmate, the referring psychologist, and a telehealth coordinator; • Primarily focused on medical management; • Each consultation lasted between 10 and 30 minutes;	Inmates’ perceptions of their telehealth experiences;
McCue et al 1997 [43] U.S.	Observational study	n = 165 telemedicine consults; Control group: n = 73 face-to-face consults;	NR	Data collected from administrative staff at the correctional centre and review of records;	Implementation of a telemedicine clinic for treatment of HIV-positive inmates: • The sessions involved taking the patient history and performing the physical examination using cameras and endoscopes to view skin lesions, examine the mouth and ears, listen to heart and lung sounds, and observe the patient walk;	Cost savings in aspects of transportation and medical payment, associated with implementation of telemedicine;
McCue et al 1998 [44] U.S.	Observational study	n = 290 telemedicine consults	NR	Data collected from the nursing staff at the correctional centre and review of medical billing data;	Implementation of a telemedicine program for provision of HIV, cardiology and oral surgery services;	Cost savings in aspects of transportation, litigation and medical, associated with implementation of telemedicine;
McCue et al 2000 [45] U.S.	Longitudinal study	n = 24 telecardiology consults during the first year; n = 78 over the second year; n = 86 during the third year;	NR	Data collected from the administrative staff at the correctional centre and review of medical billing data;	Implementation of telecardiology program: • Consults were conducted by a remote physician with the patient; • The consults involved taking the patient history and performing the physical examination using cameras to observe the patient and the digital stethoscope to listen to heart and lung sounds. Chest X-rays were evaluated by television; • EKG’s, chest X-ray reports, ancillary test results and medical records were shared by facsimile;	Cost savings in aspects of transportation and medical, associated with implementation of telecardiology;
McDonald et al 1999 [46] U.S.	Observational study	n = 1,321 teleconsultations	***Setting*:** n = 4 Federal prisons; housing predominantly male prisoners with daily average of 1,037 to 1,450; including low-, medium- and maximum-security prisoners;	Relevant data were collected from the following sources: • The Bureau of Prisons (BOP) management information and accounting system; • Telemedicine site coordinators; • Telemedicine prime contractor; • Interviews with health services administrators and clinicians;	Implementation of telemedicine program for various specialty care	Cost savings related to implementation of telehealth;
Mekhjian et al 1996 [47] U.S.	Case report	n = 69 inmates for project evaluation	NR	Completion of patient satisfaction surveys;	Implementation of telemedicine project;	Level of satisfaction of inmates
Mekhjian et al 1999 [48] U.S.	Observational study	n = 299 inmates were surveyed (n = 221 used for analysis);	***Gender*:** n = 203 males; n = 18 females; ***Setting*:** n = 31 from the maximum-security prison; n = 109 from other institutions;	Completion of a 14-item satisfaction questionnaire following each consultation;	Implementation of telemedicine services (various specialties): • Patients from the maximum-security prison: on-site teleconsultation with the specialist; a nurse and the institution doctor attended consultation with the patient; • Patients from other institutions: to be transported to the clinic with telemedicine system installed; a nurse from the clinic attended consultation with the patient;	Level of satisfaction of inmates; Impact of the contextual factors of location and physician specialty type on satisfaction levels;
Morey et al 2018 [61] U.K.	Observational study	n = 2 facilities	***Setting*:** both facilities belonged to the prison estate in the North East of England; *Facility 1 (piloted UOBBVT)*: male remand prison taking ~7000 receptions awaiting trial annually; *Facility 2 (piloted TC HCV treatment clinics)*: male prison with a stable population of 1354 inmates, with many undertaking medium lengths of sentence;	Prospective collection of clinical data; Completion of a short satisfaction questionnaire by inmates;	Implementation of UOBBVT and TC HCV treatment clinics;TC HCV treatment clinics: • Use of consultant-led TC with viral hepatitis nurse-led prison in-reach clinics; • Initial consultation with the nurse on a weekly basis: collecting inmate history and conducting a physical examination, pre-treatment blood tests, liver ultrasound and transient elastography; • Second consultation with the hepatology consultant via TC video link on a fortnightly or monthly basis (after test results became available): decisions about treatment; • All patients were then discussed in the regional hepatitis C multidisciplinary meeting and commenced the recommended DAAs;	Change of HCV RNA positive referral, attendance and antiviral treatment rates; Level of satisfaction;
Morgan, Patrick & Magaletta 2008 [49] U.S.	Quasi-experimental	n = 186 inmates Face-to-face psychological services: n = 50; Telemental health psychological services: n = 36; Face-to-face psychiatric services: n = 50; Telemental health psychiatric services: n = 50;	***Age*:** mean = 31.8 years (SD = 9.4); ***Gender*:** all males; ***Ethnicity*:** 50% Caucasian; 22.6% African-American; 21.5% Hispanic; ***Medical conditions*:** 74% suffered from mood disorders; 19% had schizophrenia or other psychotic disorders; ***Others*:** had served a median of 48 months (mode = 36 months); an average of 10.89 years of education (SD = 1.9); 36% convicted of a violent crime and 58.6% nonviolent crime;	Completion of three questionnaires on one occasion;	• Inmates were allocated to the two different modalities (face-to-face or telemental health) and for the services (psychology or psychiatry) that was considered clinically necessary; • Each psychological session lasted 30 minutes, with a general focus on issues related to adjustment and mental health stability; facilitated by one psychologist; • Each psychiatric session lasted 20 minutes, with a general focus on symptom management; facilitated by one psychiatrist;	Inmates’ perceptions of telemental health services, including: • Level of satisfaction (measured by CSQ-8); • Working alliance (measured by WAI); • Post-session mood/session evaluation (measured by SEQ);
Myers et al 2006 [50] U.S.	Observational study	n = 115 incarcerated youths	***Age and gender*:** aged between 13–15 years (n = 26 males; n = 7 females) and 16–19 years (n = 61 males; n = 21 females); ***Medical conditions*:** an average of 2.4 disorders diagnosed per youth; 20% did not receive prescribed medications; ***Setting*:** a minimum-security juvenile correctional facility located in rural area; average sentence = 6 months (ranging from 30 days to 4 years);	Review of clinical records; Completion of a satisfaction questionnaire at post consultation;	Implementation of telepsychiatry service: • The telepsychiatrist performed diagnostic evaluations, needs assessment, initial treatment, and brief follow-up; • On-site staff provided ongoing care and frequently consulted with the telepsychiatrist by phone; • Youths attended their sessions with various facility staff initially; and later they were only accompanied by the Nurse Practitioner; • 60-minute evaluation; 16 hours of consultation per month;	Level of satisfaction of the participants;
Nelson, Zaylor & Cook 2004 [51] U.S.	Observational study	n = 62 inmates	***Age*:** half of the participants aged under 30 years; ***Gender*:** 91% males; ***Ethnicity*:** 84% Caucasian; ***Setting*:** a rural county jail with 140 beds;	SCL-90-R was completed by inmates prior to attending telepsychiatry sessions; A standard evaluation form and CGI were completed by the psychiatrist following each consultation;	Implementation of a telepsychiatry clinic: • Patients screening and appointments scheduling were conducted by the jail medical officers; • Psychiatric consultations focussed on both emergency care (i.e. suicide watch) and long-term care; • Correctional officers attended both assessment and follow-up appointments; • Monthly clinic and as needed;	Agreement between psychiatric evaluation via telehealth and patient self-reported symptoms;
Rappaport et al 2018 [52] U.S.	Observational study	NR	***Setting*:** a state-wide correctional system encompassing 24 facilities with over 22,000 inmates;	Prospective collection of the number of telehealth encounters;	Enhanced telemedicine program (10 specialties): • Non-emergent consultations with specialists over telemedicine were determined by the on-site primary care physician; • Initial patient telemedicine encounters were conducted with the presence of the on-site physician; • The patient was transported to the outside medical facility if subsequent procedures were indicated; • Follow-up appointments were scheduled over telemedicine;	Number of months required to reach the break-even point;
Seol, Park and Kim 2018 [59] South Korea	Observational study	n = 406 prisoners	***Age*:** mean 39.0 years; ranging from 17 to 76 year; ***Gender*:** 91.4% males;	Retrospective review of medical records;	Teledermatological consultations once a week: • Appointments arranged via the prison officer; • At consultation, the dermatologist made diagnosis and recommended treatment based on real-time consultation and visual inspection using the LITD equipment; • Patients were transferred to the hospital if laboratory tests or a dermatologic examination were required;	Clinical improvement at follow-up consultations;
Sherwood et al 2018 [19] U.S.	Observational study	n = 376 prisoners	***Age*:** mean = 42.3 (±13.2) years; ***Gender*:** all male prisoners; ***Setting*:** average driving distance from the prison to the hospital = 186 (±151) miles (ranged 3 to 311 miles);	Retrospective review of medical records;	Telemedicine consultations in addition to in-person visits for urological care: • Telemedicine was conducted using teleconferencing and videoconferencing; • Basic physical examination was performed before or during the telemedicine visit by a primary care provider at the prison; • An on-call staff urologist for complaints or urgent conditions;	Consistency of telemedicine and in-person diagnoses; Compliance with radiologic and medication orders; In-person visits saved with telemedicine;
Taylor et al 2018 [57] Australia	Case study	n = 3,539 inmate appointments	***Setting*:** 11 correctional facilities in Queensland;	Retrospective review of patient activity;	Potential substitution of face-to-face consultations with telehealth consultations;	Modelled cost savings associated with telemedicine usage from a government perspective;
Young et al 2014 [53] U.S.	Observational study with historical controls	n = 514 in the pre-telemedicine group; n = 687 in the telemedicine group;	***Age*:** ≥18 years of age; ***Medical conditions*:** HIV-infected patients;	Data for the first six visits were collected from the pre-telemedicine and telemedicine clinic databases;	Implementation of telemedicine clinic for HIV care: • Involved a board-certified infectious disease physician, an infectious disease-trained pharmacist, and a case manager; • Both the patient and correctional nurse participated;	Effectiveness of using telemedicine for HIV care, in areas of: • Virologic suppression; • Mean CVL, mean in-care HIV load at the final visit, and CD4 T-lymphocyte counts;
Zaylor, Melson & Cook 2001 [54] U.S.	Pre-post	n = 45 inmates	***Age*:** n = 22 (49%) aged under 30; n = 11 (24%) aged between 30 and 39; n = 12 (27%) aged 40 or above; ***Gender*:** n = 41 (91%) males; n = 4 (9%) females; ***Ethnicity*:** n = 38 (84%) Caucasian; n = 7 (16%) African-American; ***Medical conditions*:** various psychiatric diagnoses, with substance dependence, abuse or withdrawal being the most common (53%); ***Setting*:** a rural county jail;	Completion of SCL-90-R by inmates on three occasions (i.e. before meeting the psychiatrist and twice during the treatment); Completion of CGI by the psychiatrist following each visit;	Implementation of telepsychiatry services: • Once-weekly scheduled clinic in addition to ad hoc consultations; • Patients screening and appointments scheduling were conducted by the jail medical officers; • Psychiatric consultations focussed on both emergency care (i.e. suicide watch) and long-term care; • Correctional officers attended both assessment and follow-up appointments;	Impact of telepsychiatry service on symptom improvement from both the patients and clinicians perspectives;
Zincone, Doty & Balch 1997 [55] U.S.	Observational study	NR	***Setting*:** 80 miles between the prison and the hospital;	NR	Implementation of telemedicine	Number of consults required to reach the break-even point; Number of years required to pay back the fixed and variable costs for telemedicine;
Zollo et al 1999 [56] U.S.	Observational study	n = 4,396 outpatient visits for cost analysis	***Setting*:** correctional facilities across Iowa;	NR	Implementation of telemedicine program, including various specialty clinics	Cost comparison between telemedicine and on-site visits;

**Abbreviations:** C-GHQ-12 = Chinese version of the 12-item General Health Questionnaire; CGI = Clinical Global Impression Scale; CSQ-8 **=** Client Satisfaction Questionnaire; CVL = Community HIV viral load; DAAs = direct-acting antivirals; EKG = Electrocardiogram; ER = Emergency room; HCV = Hepatitis C virus; HIV = Human immunodeficiency virus; I-GES = Intervention Group Environment Scale; IPP = Individual Programme Plan; LDL = Low-density lipoprotein; LITD = Live interactive teledermatology; MOCTS = Measure of Criminogenic Thinking Styles; PICTS = Psychological Screening Inventory of Criminal Thinking Styles; SCL-90-R = Symptom Rating Checklist-90-Revised; SD = Standard deviation; SEQ = Session Evaluation Questionnaire; SVR = Sustained virological response; TC = Telemedicine clinics; UOBBVT = Universal offer of blood borne virus testing; VC = Videoconferencing; WAI = Working Alliance Inventory; YDC(s) = Youth development centre(s).

### Methodological quality

The assessment of methodological quality was undertaken using the modified version of McMaster CAT for quantitative studies [28]. The study quality ratings are summarised in S2 Table. All studies clearly stated the study purpose. Majority of the studies reviewed relevant background literature and justified the need of their studies. While most studies justified their sample size, only some described their sample in detail. The psychometric properties of outcome measures were mostly ‘not addressed’, with two studies scored for reliability [39, 42] and five studies scored for both reliability and validity [34, 48, 49, 51, 58]. Over half of the included studies described their telehealth interventions in detail. Contamination and co-intervention were mostly ‘not applicable’ due to inclusion of only one group under the study. Of the studies included more than one group, only one study scored for ‘co-intervention was avoided’ [49]. Furthermore, half of the included studies did not report on drop-outs.

### Impacts and outcomes of telehealth interventions

This systematic review identified a range of outcomes as a result of implementing telehealth interventions in prison. Given the plethora of outcomes reported, and to ensure ease of interpretation, these outcomes were categorised into two overarching categories: ***Process-related outcomes*** and ***Telehealth outcomes***. The category ***Process-related outcomes*** encompassed findings related to the process of delivering healthcare within prison systems. Under this category, the findings were further merged into three sub-categories: *patient-related*, *access to healthcare* and *miscellaneous*. The category ***Telehealth outcomes*** included measurements regarding clinical- and practice-related outcomes. Additionally, costs associated with implementation of telehealth were included in this category. A summary of measurements regarding the impacts of, and outcomes from, telehealth delivered in prisons is described in Tables 3 and 4.

**Table 3:** Process-related outcomes.

Study, year and design	Patient-related					Access to healthcare				Miscellaneous
	Overall satisfaction	Therapeutic alliance	Attendance and treatment completion	Intervention effectiveness	Session evaluation–post-session mood	Waiting time from referral to treatment	Outpatient visits	Emergency room visits	Inpatient visits	
Batastini, 2016 [34] (Quasi-experimental)	**↓ & -**(VC vs in-person)	**↓ & -**(VC vs in-person)	**Treatment completion ↔**(VC vs in-person)	**↓ & -**(VC vs in-person)						
Brodey, 2000 [35] (Non randomised control)	**↔** (TC vs in-person) **+** between “*good”* and “*very good”*									
Cheng, 2018 [58] (Case control)	**+**									
Fox, 2007 [38] (Pre-post, quasi-experiment)						**↓ & +**	**↑ & +**	**Mixed**	**Mixed**	
Jimenez-Galan, 2019 [60] (Observational study)	**+**									
Magaletta, 2000 [42] (Observational study)	**+**									
McDonald, 1999 [46] (Observational study)						**↓ & +**				**Quality of care↑ & +** **Break-even point +** Less than 2 years to recapture the total capital investment
Mekhjian, 1996 [47] (Case report)	**+**									
Mekhjian, 1999 [48] (Observational study)	**+**									
Morey, 2018 [61] (Observational study)	**+**		**Attendance rate ↑ & +**							
Morgan, 2008 [49] (Quasi-experimental)	**↔** TC vs in-person	**↔** TC vs in-person			**↔**TC vs in-person					
Myers, 2006 [50] (Observational study)	**+**									
Rappaport, 2018 [52] (Observational study)										**Break-even point +** n = 32 months to recapture the capital investment
Sherwood, 2018 [19] (Observational study)										**Transportation for healthcare+** at least one fewer in-person visit could be saved for 80% - 94% patients
Zincone, 1997 [55] (Observational study)										**Break-even point +** during year 4 to pay back the total investment
Zollo, 1999 [56] (Observational study)										**Break-even point +** n = 2475 televisits per year to approach the cost of an on-site visit

***Keys*:** ↑ = increase; ↓ = decrease; ↔ = no difference; + = positive change/finding; - = negative change/finding;

***Abbreviations*:** TC = teleconsultation; VC = videoconferencing.

**Table 4:** Telehealth outcomes.

Study, year and design	Clinical-related							Practice-related		Cost
	Mental and psychological health	Criminal thinking	Establishment and achievement of goals	Diabetes-related parameters	HIV-related parameters	HCV-related parameters	Dermatology related	Diagnostic concordance	Order compliance	
Batastini, 2016 [34] (Quasi-experimental)	**↔** (VC vs in-person)	**↔** (VC vs in-person)								
Brunicardi, 1998 [36] (Case report)										**↓ & +** (with increased TC use)
Cheng, 2018 [58] (Case control)	↑* & + (TC vs control) **+** (no adverse events)									
Fox, 2006 [37] (Pre-post, quasi-experiment)										**↑ & -** (all cost measures) **Mixed** (with increased TC use)
Fox, 2008 [39] (Pre-post)			↑* & + (Year 2 vs Year 0)							
Jameson, 2008 [40] (Observational study)				**Glycaemic control** **+**						
Jimenez-Galan, 2019 [60] (Observational study)						**SVR** **+** **Other parameters** **+**				
Kassar, 2017 [41] (Observational study)				**Glycaemic control** **+** **Blood pressure** **↓ & +** **LDL profile** **↓ & +**						
McCue, 1997 [43] (Observational study)										**↓ & +**
McCue, 1998 [44] (Observational study)										**↓ & +**
McCue, 2000 [45] (Longitudinal study)										**↓ & +** (with increased TC use)
McDonald, 1999 [46] (Observational study)										**↓ & +**
Morey, 2018 [61] (Observational study)						**SVR** **+** **Treatment rate** **↑ & +**				
Nelson, 2004 [51] (Observational study)								+*		
Seol, 2018 [59] (Observational study)							**+**			
Sherwood, 2018 [19] (Observational study)								**+**	**+**	
Taylor, 2018 [57] (Case study)										**↓ & +**
Young, 2014 [53] (Observational study with historical controls)					**Virologic suppression** ↑* & + (p<0.001) **Mean CD4 counts** ↑* & + **Virologic burden** ↓* & + (p<0.001)					
Zaylor, 2001 [54] (Pre-post)	↓* & +									

***Keys*:** ↑ = increase; ↓ = decrease; ↔ = no difference; + = positive change/finding;— = negative change/finding; *statistically significant (p<0.05 or otherwise specified);

***Abbreviations*:** CVL = community HIV viral load; HbA1c = Haemoglobin A1c; HCV = hepatitis C virus; HIV = Human immunodeficiency virus; LDL = low density lipoproteins; SVR = sustained virological response; TC = teleconsultation; VC = videoconferencing.

#### Process-related outcomes

A total of 16 included studies reported on process-related outcomes. Of these, 10 studies reported on findings associated with the sub-category *patient-related* [34, 35, 42, 47–50, 58, 60, 61], two studies explored findings relative to the sub-category *access to healthcare* [38, 46] and five studies measured outcomes which were categorised under the sub-category *miscellaneous* [19, 46, 52, 55, 56].

#### Process-related outcomes: Patient-related

This sub-category explored participants’ perspectives on the process of prison telehealth delivery. Ten studies commonly evaluated on level of satisfaction with telehealth [34, 35, 42, 47–50, 58, 60, 61], two studies investigated therapeutic alliance [34, 49], two studies explored treatment attendance and completion status [34, 61] with one of them further compared features affecting intervention effectiveness [34], and another study measured post-session mood as means of evaluating telehealth sessions [49].

Overall, the findings were mixed in terms of participants’ satisfaction with telehealth delivered in prisons. Seven studies reported that participants were generally satisfied with the delivery of telehealth consultations. In particular, Cheng and colleagues [58] suggested a favourable response to telepsychiatry amongst participants, as indicated by a mean satisfaction score of 16.48 and standard deviation of 4.35, with possible scores ranging from 9 (the most satisfied) to 45 (the least satisfied). Both Morey et al [61] and Mekhjian et al [47] found that majority of their study participants were satisfied with telehealth consultations (80% rated *“good”* or *“excellent”* and 51 out of 69 participants indicated a satisfaction, respectively). Mekhjian and colleagues [47] further reported that their satisfied participants also felt comfortable asking questions.

In addition to assessing overall satisfaction, some studies evaluated various aspects of patient-physician interaction via telehealth. For example, Jimenez-Galan and colleagues [60] sought feedback on technical quality of image and sound, comfortability, perceived professionalism as well as overall assessment from both participating prisoners and doctors. They discovered a high level of satisfaction with at least 67% rated *“good”* or *“very good”* on each of these components and all participating doctors expressed a preference for teleconsultation over the conventional approach. Consistently, another study indicated an overall satisfaction with teleconsultations in aspects of both information exchange and patient comfort [48]. The study further investigated the impact of contextual factors, including location of prisoners and type of healthcare specialties, on satisfaction levels. There was a significantly higher satisfaction level on the patient comfort aspect amongst prisoners who did not require transportation when attending telehealth consultations (p<0.01). On the other hand, while Myers and colleagues [50] also found an overall satisfaction with telepsychiatry consultations, the incarcerated youths in this study were less satisfied with the presence of staff during consultations due to privacy concerns. Magaletta, Fagan and Peyrot [42] suggested a positive change of inmates’ perceptions on telehealth over time, in addition to positive initial ratings. Interestingly, the authors further noted a significant difference in the distribution of scores for positive response between the audio and visual questions (p = 0.017), highlighting that the audio quality appeared to be a critical factor contributing to the participants’ overall perceptions of telehealth. Furthermore, when compared to previous in-person psychiatric treatment experience off-site, majority of the participants felt that telehealth was comparable or better than the in-person treatment.

Another three studies evaluated telehealth consultations in comparison to in-person visits. Studies conducted by Brodey et al [35] and Morgan, Patrick and Magaletta [49] revealed comparable satisfaction levels with mental health services between telehealth and in-person groups, with Brodey et al [35] found an averaged rating between *“good”* and *“very good”* for both modalities. In contrast to these findings, Batastini and Morgan [34] indicated a generally less satisfaction level amongst participants who received telepsychology services compared to those who attended in-person consultations, albeit insignificant.

Two studies measured the therapeutic alliance between patient and therapist in terms of development of goals, reaching goals and quality of relationship [34, 49]. While both studies reported lack of significant differences between participants receiving telehealth and in-person mental health services, Batastini and Morgan [34] suggested that participants from the telepsychology group appeared to be less trusting and accepting of their therapist and had lower level of agreement on the goals and tasks than their counterparts who received face-to-face services.

In addition to the working alliance, Batastini and Morgan [34] also compared treatment completion status and features relating to the intervention between telehealth and in-person groups. In particular, there were no statistically significant differences in treatment completion status between the two groups. However, the participants who received telepsychology tended to be less cohesive and experienced more counterproductive activity within the group. They also felt the implementation was less organised and the therapist was less prepared, compared to their peers in the in-person group. In contrast, Morey and colleagues [61] suggested a positive, noticeable increase of attendance rate for their HCV treatment clinic (50% at pre and 83% at post implementation of telehealth).

Furthermore, participants’ post-session mood was assessed by one study as means of evaluating telemental health sessions [49]. The findings revealed that there were no statistically significant differences in participants’ evaluation (including session depth, smoothness, positivity and arousal) between telehealth and in-person groups.

#### Process-related outcomes: Access to healthcare

A total of two included studies discussed the impact of implementing prison telehealth interventions on patients’ access to healthcare services [38, 46]. The findings from both studies highlighted an overall reduction of waiting times from referral to treatment. In particular, Fox and colleagues [38] reported a 50% and 57% decrease of the average number of waiting days in the first and second year of telehealth implementation compared to pre-implementation, respectively, despite more patients were referred and seen for diagnoses (47% increase in the first year and 59% in the second year).

Additionally, Fox, Somes and Waters [38] further explored the effectiveness of telehealth implementation on improving outpatient, emergency department (ED) and inpatient visits. Overall, outpatient visits increased by 40% during the two years of implementation, with a statistically significant increase of outpatient visits at three of the four adolescent detention facilities. While the changes of ED and inpatient visits were mixed across the facilities, three out of the four facilities remained unchanged for ED visits and a declining trend was discovered for inpatient visits. Further analysis also revealed that each 1% increase in utilisation of telehealth was associated with 1% increase of outpatient visits and 7% reduction of ED visits.

#### Process-related outcomes: Miscellaneous

Break-even point was a commonly reported finding under this sub-category. As the term suggests, the focus of this outcome related to the frequency and duration of telehealth utilisation commensurate to the original capital expenditure. A total of four included studies analysed this outcome [46, 52, 55, 56]. Overall, the duration to reach the break-even point was varied across these studies, with a timeframe from less than two years [46] to 32 months [52] or during the fourth year of telehealth implementation [55]. Zollo and colleagues [56] reported that in order to achieve the break-even costs for one telehealth visit vis-à-vis one face to face visit, approximately 2,475 annual telehealth visits were required.

One study evaluated on use of telehealth for urological care amongst male prisoners [19]. In particular, the study estimated that there was at least one fewer in-person visit to be avoided for 80% to 94% of the study participants as a result of using telehealth in prison. Furthermore, another study [46] reported that implementation of prison telehealth was also associated with improved quality of care, such as increased access to more experienced specialists and specialty care which was not locally available.

#### Telehealth outcomes

A total of 19 included studies reported on telehealth outcomes. Of these, seven studies analysed costs associated with use of prison telehealth compared to pre-implementation or in-person consultations [36, 37, 43–46, 57], ten studies investigated outcomes from a clinical perspective, these included mental and psychological health, criminal thinking, establishment and achievement of goals, diabetes, HIV and HCV related parameters, as well as dermatology associated outcomes [34, 39–41, 53, 54, 58–61] and two studies reported on findings in relation to health professionals practice as a result of implementation of prison telehealth [19, 51].

#### Telehealth outcomes: Cost

The findings on costs associated with implementation of telehealth in prisons were mixed. Fox, Somes and Waters [37] indicated an overall increased service utilisation across all cost measures after using telehealth, suggesting increased cost associated with telehealth. Of the cost measures, there were significant increases in *average medical cost per student per month* (47.63%, p<0.01), *total cost per student per month* (43.97%, p = 0.01) and *medical cost per encounter* (34.58%, p<0.01), with greatest increases in areas of *outpatient cost per centre per month* (53.76%) and *emergency room cost per centre per month* (149.71%). However, upon analysis of the relationship between the level of telehealth utilisation at individual youth development centre and associated cost, a negative relationship was discovered across most cost measures at more than one centre. A similar finding was reported by another study [45], in which the cost associated with telecardiology consultations was higher than the in-person cardiology service during the first year, but cost savings occurred with increased use of telecardiology in the second and third year of implementation. The direct impact of increased utilisation of telehealth on the cost savings was also supported by another study [36].

Another four studies revealed benefits at post implementation of telehealth [43, 44, 46, 57]. In particular, a cost-benefit analysis in McCue et al [43] showed a cost saving per televisit compared to in-person clinic, as a result of the net benefit by using telehealth for management of HIV-positive prisoners over the seven-month study period. A follow-up study supported this finding and reported a net saving per visit of using telehealth compared to conventional care for the next 12 months of implementation [44]. Similarly, cost savings were also reported in the study conducted by McDonald and colleagues [46] as a result of averted trips (to local specialists) and transfers (by aircraft) associated with using telehealth. Based on the utilisation pattern, the authors anticipated a less costly or a comparable cost of an operational telehealth system as opposed to conventional care in prisons. Another consistent finding was discovered in an Australian study, which modelled the cost consequence as a result of substituting in-person specialist outpatient consultations with teleconsultations [57]. With the telehealth infrastructure already in place, use of telehealth would result in cost savings up to $969,731 Australian dollars in a 12-month period, depending on the level of utilisation.

#### Telehealth outcomes: Clinical-related

Of the studies which used telehealth for mental health services, two of them used the Symptom Checklist-90-Revised (SCL-90-R) measuring clinical outcomes in relation to psychological symptoms and related distress [34, 54]. One study found that the participants perceived less distress during telepsychiatry treatment, as indicated by decreased self-rated symptom scores over time (p<0.05), which was in agreement with the psychiatrist impression [54]. However, the other study reported no significant differences in mental health functioning between telehealth and in-person groups [34]. This study further highlighted comparably low occurrence of disciplinary infractions and mental health-related sick call requestions [34]. These findings were partly supported by another study [58]. In particular, the authors evaluated pre-post change of psychological health between the intervention and control group participants by using the Chinese version of the 12-item General Health Questionnaire (C-GHQ-12). The results showed a significantly higher pre-post difference in C-GHQ-12 score of the intervention group, compared to the control group (p = 0.023). The authors also indicated a moderately strong and positive but insignificant association between pre-post difference in C-GHQ-12 score of the first and second teleconsultations (r = 0.309, p = 0.103), suggesting a sustainable effect on psychological health between telepsychiatry sessions. They further highlighted that there were no significant adverse events associated with using telepsychiatry.

Furthermore, two studies assessed change of criminal thinking and goal establishment and achievement resulted from using telehealth for mental and behavioural care. While one study highlighted no significant difference in criminal thinking between telehealth and in-person groups [34], the other study revealed a significant increase in the total number of goals established by adolescents in Year 2 of telehealth implementation compared to pre-implementation (p<0.05), with significantly higher number of goals in the areas of education, family, health and social skills (p<0.05). Additionally, the proportion of youths who were able to achieve their goals also appeared to be affected by the presence of telehealth. In particular, there were significant increases in the proportion of youths attaining goals in the family, health and social skills areas (p<0.05) at second-year post implementation compared to pre-implementation [39].

Two included studies assessed diabetes-related parameters in terms of glycaemic control, blood pressure and low-density lipoproteins (LDL) profile after receiving treatment via telehealth. Jameson and colleagues [40] reported that the number of participants who had a poorer control of haemoglobin A1c (HbA1c; HbA1c > 9%) was declined by 40% at their final visit, with 29% achieved the goal HbA1c (i.e. <7%). They further suggested that participants who had greatest improvements in HbA1c appeared to have more televisits (mean = 4.0 compared to 2.7 visits), were consulted more frequently (30% compared to 20% for every four months) and had longer follow-up (mean = 12.8 compared to 7.9 months). Consistently, Kassar, Roe and Desimone [41] reported a reduction in mean HbA1c as a result of using telehealth for diabetes management, with over half (56.9%) of the analysed individuals (n = 58) had improvements in their HbA1c levels. The study further observed positive findings associated with reductions in both mean blood pressure and mean LDL amongst the participants at their final televisits.

Clinical outcomes in relation to management of HIV infected prisoners using telehealth were reported by one study [53]. HIV subspecialty care managed by a multi-disciplinary team via telehealth was associated with significantly greater proportion of individuals with complete virologic suppression (p<0.001), higher mean CD4 counts (p = 0.032) and significantly (p<0.001) lower means in community HIV viral load (CVL) and in-care HIV load.

Additionally, integrating telehealth into HCV care process within the prison system also appeared beneficial as reported by two studies [60, 61]. Both studies highlighted a high sustained virological response (SVR) achieved by participants, with one study reported an overall SVR rate of 96.9% [60] while another achieved 100% SVR [61]. Positive outcomes, such as successful rescue of all virological failures with a second direct-acting antivirals regime, prevention of HCV reinfection cases, and significant improvement in liver stiffness at one-year post implementation (p<0.001) were further discovered by Jimenez-Galan and colleagues [60]. Similarly, Morey and colleagues [61] revealed encouraging outcomes when compared pre and post implementation. In particular, 71% (n = 57) participants started anti-HCV treatment with 73% of them completed the treatment while in prison, compared to four people who commenced the treatment prior to the implementation of telehealth.

Only one included study primarily evaluated the effect of teledermatological consultations for prisoners [59]. In this study, while 86.7% participants who attended a follow-up consultation showed clinical improvement of their dermatologic conditions and only a small proportion had no change or aggravated or recurred outcomes, the authors highlighted limitation associated with a relatively low follow-up rate (38.2%).

#### Telehealth outcomes: Practice-related

Diagnostic concordance and order compliance were measured by two included studies [19, 51]. Nelson, Zaylor and Cook [51] found a significant, positive correlation between overall psychiatric ratings (evaluated by the psychiatrists) and inmate self-report of overall symptoms (p<0.05). This finding was supported by Sherwood and colleagues [19], who reported 90% concordance between telehealth and in-person diagnoses, with no disagreement of malignancy diagnosis. The authors further indicated high compliance with radiologic (91%) and medication (89%) orders through telehealth [19].

### Summary of evidence

Building upon a moderate body of research evidence encompassing a myriad of quantitative research designs and methodological quality, this systematic review has identified mixed evidence on the impact of, and outcomes from, telehealth in prisons. Collectively, the evidence base does highlight some positive impacts of telehealth, which at the least, is as effective as conventional care while achieving patient satisfaction. However, due to methodological flaws (such as small sample size, lack of psychometric properties of outcome measures) and heterogeneity amongst the included studies (including variability in study populations, types of healthcare services, intervention parameters and outcome measures), care should be taken in interpretation and application. Furthermore, given that only a handful of studies reported data for some outcomes (such as clinical-and practice-related outcomes), unequivocal recommendations cannot be made for these outcomes.

## Discussion

Health problems experienced by prisoners are more severe than the general community which is compounded by barriers to timely access to healthcare services. While telehealth may have a role to play in addressing this, to date there has been limited focus on systematically synthesising the evidence base on the effectiveness of telehealth for prisoners. By addressing this knowledge gap, a range of impacts and outcomes associated with implementation of prison telehealth were identified from the included studies. These findings were categorised into two overarching categories–*process-related outcomes* and *telehealth outcomes*.

The popularity of telehealth is often purported due to its ability to improve access to health services while remaining efficient in terms of resources required [20]. Given this to be the case, many included studies did explore the efficiency of telehealth in prisons through break-even points and cost consequences compared to conventional approaches, from the perspectives of healthcare and correctional systems. While some studies revealed that telehealth to be less costly compared to in-person consultations, others indicated, not unexpectedly, a higher initial cost with a trend of reduction over time as the use of telehealth increased, which is consistent with other literature [20]. Interestingly, Wade and colleagues [20] further indicate that when solely evaluated from the perspective of health services, the number of studies reporting telehealth to be cost saving reduced dramatically. Whereas when telehealth was assessed from the patient perspective, cost savings were commonly identified as a result of savings in patient travel costs [20, 62].

Patient-related outcomes, particularly patient satisfaction and clinical outcomes were another aspect measured by many studies. In terms of patient satisfaction, most studies reported an overall satisfaction with telehealth or comparable satisfaction levels between telehealth and in-person treatments, such as for psychiatry care. This finding is largely consistent with other literature which have investigated patients’ overall satisfaction with telehealth in areas such as psychiatry, dermatology and multi-specialty services [63, 64]. Patient-related clinical outcomes associated with telehealth have been previously assessed by several studies. For example, in a study conducted by Tuerk and colleagues [65], statistically significant reductions in self-reported post-traumatic stress disorder (PTSD) symptoms were reported by patients treated via telehealth which was comparable to traditional in-person treatment. However, not all literature supports the positive impacts of telehealth on patient outcomes. A literature review evaluated both real-time and ‘store-and-forward’ modalities used in various fields of healthcare services (such as pathology, radiology and different specialties) and reported equivocal evidence related to clinical management and outcomes of telehealth [63]. Similarly, another literature review conducted by Hersh and colleagues [66] reported inconclusive evidence on clinical effectiveness of telehealth.

These mixed findings on the impact of telehealth is likely due to a range of reasons. First, is the variability in the type and manner in which telehealth services were developed and delivered across these studies. As organisational model of care is a critical feature in shaping the value of telehealth services [20], within individual correctional facilities, factors such as resources and training availability, the type(s) of specialist care needed by prisoners and the suitability of specialty consultation via telehealth may all have influenced the various models of telehealth services that were implemented and evaluated [67]. Second, the positive findings for process measures and patient satisfaction may align with what occurs at the frontline of prison telehealth as these are likely to be of most interest to those responsible for prison healthcare given lengthy wait times, limited access and poor prisoner experience are likely to be the biggest issue. While these do impact clinical outcomes, this may need longer term follow up and ongoing research. Similarly, it may take a while for costs to break even, due to higher initial set up costs, but with advancement in, and affordability of, technology, such set up costs are likely to be lesser and more affordable in the future. Finally, successful implementation of prison telehealth is likely dependent on a multitude of factors, including but not limited to the context of the commissioning systems and the interface between the prison and health systems. In a recent systematic review which explored factors influencing successful implementation of prison telemedicine, Edge and colleagues [68] highlight this very issue and acknowledge the need for comprehensive implementation strategy which involves stakeholder buy-in, recognition of local contextual enablers and barriers and balancing of anticipated benefits with adequate resourcing.

### Limitations

As with any research, there are some limitations to this systematic review. While the systematic searching of the literature identified a total of 29 quantitative studies, there are concerns with the methodological quality. These concerns are particularly in aspects such as small sample size and lack of adequate sample descriptions, gender bias towards males, variability in telehealth interventions, use of diverse outcome measures with limited reporting on psychometric properties and retrospective data collection methods (due to the observational nature of many of the included studies). Additionally, as this review did not include any qualitative research on this topic, in-depth exploration of prisoner perspectives of telehealth was not captured. Given most of the included studies were conducted in the United States of America and majority of these studies were published more than a decade ago, generalisability of these findings to a wider context is limited. Furthermore, this review only included studies published in English, which may introduce publication (language) bias in study selection due to exclusion of other relevant articles which were published in languages other than English.

## Conclusions

This systematic review provides mixed evidence on the impact of, and outcomes from, telehealth in prisons. While the evidence base does highlight some positive impacts of telehealth, which in prisons may be more effective than, or at least as effective as, conventional care, it is also important to consider the local context and drivers that may influence what, when and how telehealth services are provided. Additionally, for telehealth to fulfil its potential and achieve sustainability, critical factors encompassing seamless integration into routine practice, financial sustainability, interdisciplinary collaboration, and regular evaluation need to be addressed throughout the process. With the rapid growth of, and access to, emerging technology and its influence on telehealth, ongoing research is warranted to inform an evolving evidence base.

## Supporting information

S1 File PRISMA 2009 checklist. (DOC)

S1 Table Ovid Medline search strategy. (DOCX)

S2 Table Quality assessment for included studies. (DOCX)
