# Supplementary material for: The impacts of and outcomes from telehealth delivered in prisons: A systematic review
Source: PLoS One. 2021 May 17;16(5):e0251840. doi: 10.1371/journal.pone.0251840 (PMC8128277; doi:10.1371/journal.pone.0251840)
Supplement: S1 Table — (DOCX) [file pone.0251840.s002.docx]

**S1 Table. Ovid Medline search strategy**

| **#** | **Searches** |
| --- | --- |
| 1 | exp telemedicine/ or remote consultation/ or telepathology/ or teleradiology/ or telerehabilitation/ |
| 2 | exp VIDEOCONFERENCING/ |
| 3 | ((rehabilitat* or consult* or health or pathology* or radiolog* or medicine or care or monitor* or psychiatr* or audiolog* or speech or audiometr*) adj5 (virtual or remote or tele or mobile)).ti,ab,kw. |
| 4 | (telehealth or telemedicine or ehealth or mhealth or telepatholog* or teleradiolog* or telerehabilitat* or teleconsult* or telecare or telemonitor* or telepsychiatr* or teleaudiolog* or teleradiolog* or telespeech* or teleaudiometr* or e-health or m-health or videoconferenc* or video-conferenc* or video-monitor* or videomonitor* or videoconference* or video-conferenc*).ti,ab,kw. |
| 5 | 1 or 2 or 3 or 4 |
| 6 | Prisons/ |
| 7 | Prisoners/ |
| 8 | ((Penal or custodial or correction or detention) adj5 (Institution* or facility* or center$1 or centre$1)).ti,ab,kw. |
| 9 | (confined or confinement or imprison* or inmate* or incarcerat* or jail* or gaol*).ti,ab,kw. |
| 10 | 6 or 7 or 8 or 9 |
| 11 | 5 and 10 |
| 12 | "outcome and process assessment (health care)"/ or "outcome assessment (health care)"/ |
| 13 | (assess* or outcome* or measure* or eval*).ti,ab,kw. |
| 14 | 12 or 13 |
| 15 | 11 and 14 |
| 16 | limit 15 to english language |
