# Supplementary material for: The impacts of and outcomes from telehealth delivered in prisons: A systematic review
Source: PLoS One. 2021 May 17;16(5):e0251840. doi: 10.1371/journal.pone.0251840 (PMC8128277; doi:10.1371/journal.pone.0251840)
Supplement: S2 Table — (DOCX) [file pone.0251840.s003.docx]

**S2 Table. Quality assessment for included studies**

| Study & year | Quantitative Appraisal Criteria | | | | | | | | | | | | | | Total  (out of 12* or 14) |
| --- | --- | --- | --- | --- | --- | --- | --- | --- | --- | --- | --- | --- | --- | --- | --- |
|  | **Q1** | **Q2** | **Q4b** | **Q4c** | **Q5a** | **Q5b** | **Q6a** | **Q6b** | **Q6c** | **Q7a** | **Q7b** | **Q7c** | **Q7d** | **Q8** |  |
| Batastini & Morgan 2016 [34] | Y | Y | Y | Y | Y | Y | Y | NAD | NAD | Y | Y | Y | Y | Y | 12/14 |
| Brodey et al 2000 [35] | Y | Y | N | Y | NAD | NAD | Y | NAD | NAD | Y | Y | Y | Y | Y | 9/14 |
| Brunicardi 1998 [36] | Y | N | N | Y | NAD | NAD | N | N/A | N/A | N | Y | Y | N | Y | 5/12 |
| Cheng et al 2018 [58] | Y | Y | Y | Y | Y | Y | Y | NAD | NAD | Y | Y | Y | Y | Y | 12/14 |
| Fox, Somes & Waters 2006 [37] | Y | Y | N | Y | NAD | NAD | N | N/A | N/A | Y | Y | Y | N | Y | 7/12 |
| Fox, Somes & Waters 2007 [38] | Y | Y | N | Y | NAD | NAD | Y | N/A | N/A | Y | Y | Y | N | Y | 8/12 |
| Fox et al 2008 [39] | Y | Y | N | Y | Y | NAD | N | N/A | N/A | Y | Y | Y | N | Y | 8/12 |
| Jameson et al 2008 [40] | Y | N | Y | Y | NAD | NAD | Y | N/A | N/A | N | Y | Y | Y | Y | 8/12 |
| Jimenez-Galan et al 2019 [60] | Y | Y | Y | Y | NAD | NAD | Y | N/A | N/A | Y | Y | Y | Y | Y | 10/12 |
| Kassar, Roe & Desimone 2017 [41] | Y | Y | Y | Y | NAD | NAD | Y | N/A | N/A | Y | Y | Y | Y | Y | 10/12 |
| Magaletta, Fagan & Peyrot 2000 [42] | Y | Y | N | Y | Y | NAD | Y | N/A | N/A | Y | NAD | Y | Y | Y | 9/12 |
| McCue et al 1997 [43] | Y | Y | N | Y | NAD | NAD | N | NAD | NAD | N | Y | Y | N | Y | 6/14 |
| McCue et al 1998 [44] | Y | Y | N | Y | NAD | NAD | N | NAD | NAD | N | Y | Y | N | Y | 6/14 |
| McCue et al 2000 [45] | Y | Y | N | Y | NAD | NAD | Y | N/A | N/A | N | Y | Y | N | Y | 7/12 |
| McDonald et al 1999 [46] | Y | N | N | Y | NAD | NAD | N | N/A | N/A | N | NAD | Y | N | Y | 4/12 |
| Mekhjian et al 1996 [47] | Y | N | N | N | NAD | NAD | N | N/A | N/A | N | NAD | Y | N | Y | 3/12 |
| Mekhjian et al 1999 [48] | Y | Y | N | N | Y | Y | Y | N/A | N/A | Y | Y | Y | Y | Y | 10/12 |
| Morey et al 2018 [61] | Y | Y | N | Y | NAD | NAD | Y | N/A | N/A | N | NAD | Y | Y | Y | 7/12 |
| Morgan, Patrick & Magaletta 2008 [49] | Y | Y | Y | N | Y | Y | Y | NAD | Y | Y | Y | Y | Y | Y | 12/14 |
| Myers et al 2005 [50] | Y | Y | Y | Y | NAD | NAD | Y | N/A | N/A | N | Y | Y | N | Y | 8/12 |
| Nelson, Zaylor & Cook 2004 [51] | Y | Y | Y | Y | Y | Y | Y | N/A | N/A | Y | NAD | Y | N | Y | 10/12 |
| Rappaport et al 2018 [52] | Y | Y | N | N | NAD | NAD | Y | N/A | N/A | N | Y | Y | N | Y | 6/12 |
| Seol et al 2018 [59] | Y | Y | Y | Y | NAD | NAD | Y | N/A | N/A | N | NAD | Y | Y | Y | 8/12 |
| Sherwood et al 2018 [19] | Y | N | Y | Y | NAD | NAD | N | N/A | N/A | N | Y | Y | Y | Y | 7/12 |
| Taylor et al 2018 [57] | Y | Y | N | Y | NAD | NAD | N | N/A | N/A | N | Y | Y | Y | Y | 7/12 |
| Young et al 2014 [53] | Y | Y | N | Y | NAD | NAD | N | NAD | NAD | Y | Y | Y | N | Y | 7/14 |
| Zaylor, Nelson & Cook 2001 [54] | Y | Y | Y | Y | NAD | NAD | Y | N/A | N/A | Y | Y | Y | Y | Y | 10/12 |
| Zincone, Doty & Balch 1997 [55] | Y | N | N | N | NAD | NAD | N | N/A | N/A | N | Y | Y | N | Y | 4/12 |
| Zollo et al 1999 [56] | Y | N | N | Y | NAD | NAD | N | N/A | N/A | N | Y | Y | N | Y | 5/12 |
| Abbreviations: Y=Yes; N=No; N/A=Not Applicable; NAD=Not Addressed  Criteria: 1. Study purpose – was the study purpose stated clearly? 2. Literature – was the relevant background literature reviewed? 4b. Was the sample described in detail? 4c. Was the sample size justified?) 5. Outcomes (5a. Were the outcome measures reliable? 5b. Were the outcomes measures valid?) 6. Intervention (6a. The intervention was described in detail? 6b. Contamination was avoided? 6c. Co intervention was avoided?) 7. Results (7a. Results were reported in terms of statistical significance? 7b. Were the analysis methods appropriate? 7c. Clinical importance was reported? 7d. Drop outs were reported?) 8. Conclusions and clinical implications – were the conclusions appropriate given the study methods and results?  Key: * = “Not applicable” for Criteria 6b and 6c, if there was only one group under study. Thus, a total of 12 applicable criteria; | | | | | | | | | | | | | | | |
